# Supplementary material for: Hepatic-Specific FGF21 Knockout Abrogates Ovariectomy-Induced Obesity by Reversing Corticosterone Production
Source: Int J Mol Sci. 2023 Oct 5;24(19):14922. doi: 10.3390/ijms241914922 (PMC10573867; doi:10.3390/ijms241914922)
Supplement: Supplementary file 1 [file ijms-24-14922-s001.zip › New folder/Supplemental figure.pdf]

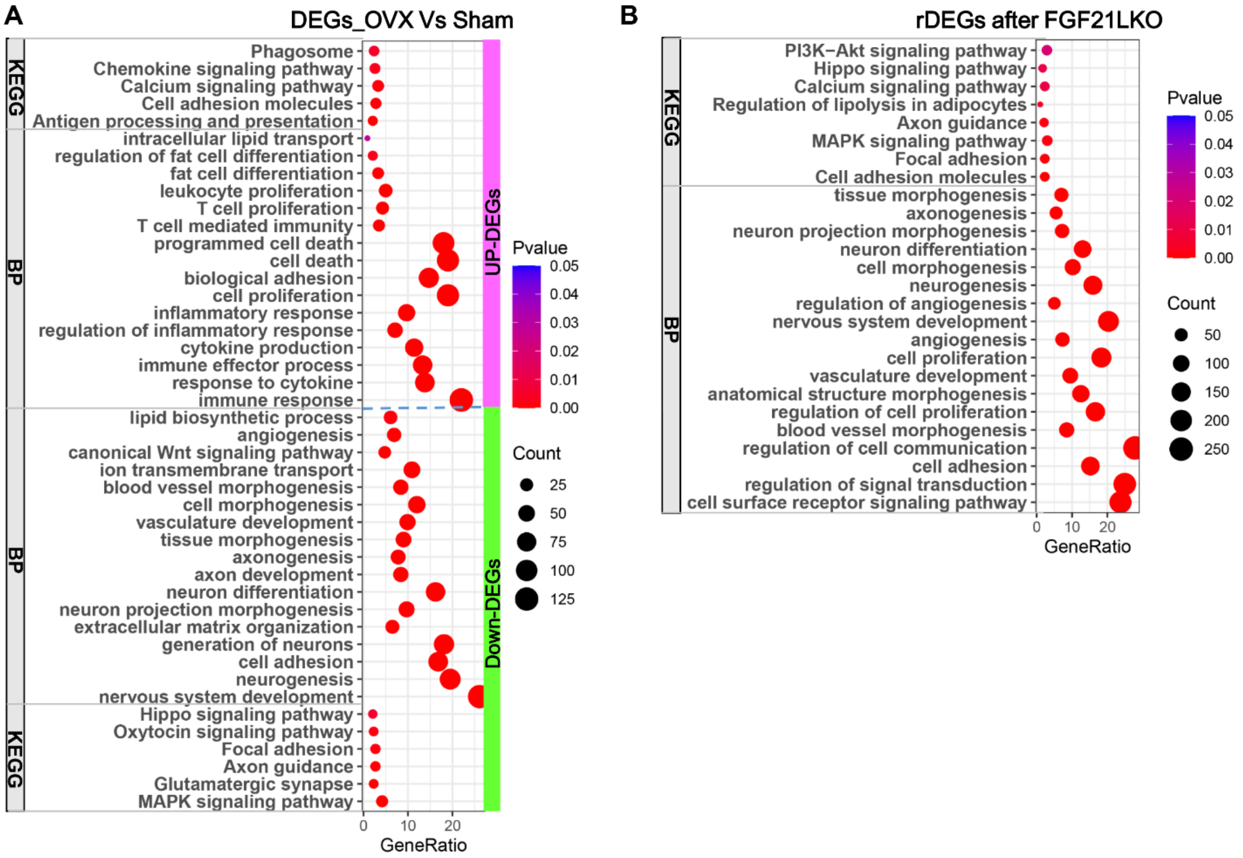

Fig. S1. Functional enrichment analysis of DEGs and rDEGs. (A) Biological process (BP) and KEGG pathway of DEGs between OVX versus Sham using DAVID enrichment analysis. (B) Biological process (BP) and KEGG pathway of rDEGs using DAVID enrichment analysis. rDEGs are genes whose expression in OVX+FGF21 LKO were significantly different from OVX but recovered back to the sham levels after FGF21 LKO.
